# Integrating palliative care within acute stroke services: developing a programme theory of patient and family needs, preferences and staff perspectives

**DOI:** 10.1186/1472-684X-11-22

**Published:** 2012-11-09

**Authors:** Christopher R Burton, Sheila Payne

**Affiliations:** 1 School of Healthcare Sciences, Bangor University, Bangor Gwynedd, LL57 2EF, UK; 2 Division of Health Research, Faculty of Health and Medicine, Lancaster University, Lancaster, Lancashire, LA1 4YT, UK

**Keywords:** Acute stroke, Palliative care, Integration, Synthesis, Theory development, End of life care

## Abstract

**Background:**

Palliative care should be integrated early into the care trajectories of people with life threatening illness such as stroke. However published guidance focuses primarily on the end of life, and there is a gap in the evidence about how the palliative care needs of acute stroke patients and families should be addressed. Synthesising data across a programme of related studies, this paper presents an explanatory framework for the integration of palliative and acute stroke care.

**Methods:**

Data from a survey (n=191) of patient-reported palliative care needs and interviews (n=53) exploring experiences with patients and family members were explored in group interviews with 29 staff from 3 United Kingdom stroke services. A realist approach to theory building was used, constructed around the mechanisms that characterise integration, their impacts, and mediating, contextual influences.

**Results:**

The framework includes two cognitive mechanisms (the legitimacy of palliative care and individual capacity), and behavioural mechanisms (engaging with family; the timing of intervention; working with complexity; and the recognition of dying) through which staff integrate palliative and stroke care. A range of clinical (whether patients are being ‘actively treated’, and prognostic uncertainty) and service (leadership, specialty status and neurological focus) factors appear to influence how palliative care needs are attended to.

**Conclusions:**

Our framework is the first, empirical explanation of the integration of palliative and acute stroke care. The specification in the framework of factors that mediate integration can inform service development to improve the outcomes and experiences of patients and families.

## Background

There is a lack of evidence developed or validated within a stroke context to help clinicians meet the palliative care needs of patients and families. Synthesising earlier research which prospectively identified stroke patients’ palliative care problems and needs [1], experiences and preferences [2] and staff perspectives, this paper provides the first theoretical explanation of how palliative care and acute stroke care can be integrated around the needs and preferences of patients and families.

Despite advances in the early identification and clinical management of patients with stroke, a significant proportion of patients die in the acute phase. Most recent estimates suggest that the 30-day mortality rate is 17% [3], although there is variation in mortality rates across stroke sub-type [4]. In the United Kingdom (UK), successive editions of national guidelines have recognised the importance of providing access to palliative care for patients at the end of life. Guidance is built on the premise that stroke teams can recognise patients who may benefit from palliative care; staff have the necessary training in the principles and practice of palliative care to support dying patients; and that access to specialist palliative care expertise is available [5].

The evidence-base for palliative care within a stroke context is limited: recommendations focus exclusively on end of life, and draw mostly on research completed in cancer populations. These may only partly be transferable to a stroke context. This paper addresses this gap by investigating the integration of palliative care into the acute stroke pathway.

Dealing with palliative care and end of life issues places considerable demands on the resilience of patients and family members. The role of health services is to provide appropriate and effective support helping patients and families to cope with, and adapt to these demands, although performance may be problematic [6]. Information provision, communication and decision-making within a multi-disciplinary team context, and in partnership with patients and family to determine preferences for care are key [7]. As with comparable health care systems, health policy in UK end-of-life care highlights the importance of developing generalist palliative care expertise outside of cancer services. Enabling patient choice about where care is delivered is expected to lead to a greater proportion of people dying at home rather than in hospital [8].

The conceptual basis for palliative care outlined by the World Health Organisation (WHO) [9] is broader than end of life care, and stresses implementation earlier in the disease trajectory in conjunction with other therapies intended to minimise disease progression and prolong life. It is now widely recognised that palliative care has a crucial role in the care received by patients and carers throughout the course of a disease process. Its supportive nature is intended to help the patient to maximise the benefits of treatment and to live as well as possible with the effects of the disease [10].

In practice, acute stroke services will be increasingly required to attend to palliative and end of life issues. Significant advances have been made in the implementation of evidence of the effectiveness of rapid neurological assessment, specialist management and organised service design for people affected by stroke. The stroke service model has shifted from one that reflects a sense of therapeutic nihilism, historically located within gerontological medicine, to one that reflects neurological urgency and optimism. Although public health initiatives and lifestyle changes may explain a general downward trend in stroke incidence [11], the development of thrombolytic therapy for acute stroke, effective secondary prevention strategies, and organised specialist services that integrate early rehabilitation [12,13] reduce the impact of stroke for patients, families and society. Clinical guidelines and health policy indicate that all stroke patients should be given the opportunity to benefit from acute stroke services. Prognostic models are not sufficiently sensitive to identify, within a practice context, patients that are likely to survive stroke [14], and there is evidence that patients with more severe strokes (defined by the Barthel Index measure of functional ability and dependence) benefit from stroke unit care [12].

A critical review of the international literature on palliative care within stroke yielded seven studies; four of which were completed within the United Kingdom [15]. No intervention studies were found. Synthesis of the studies provided the following information:

Many patients who died after stroke did not receive optimal symptom control.

Patients were not perceived to receive ‘sufficient’ help to overcome psychological problems.

Informal caregivers report difficulty accessing information about the patient’s medical condition.

The caring experience was distressful for family carers, not generally felt to be rewarding, with high reports of insufficient help and assistance.

Palliative care interventions have a role in the care of stroke patients, and should be systematically provided on the basis of need.

National Clinical Guidelines for Stroke [5] recommend that patients should have access to specialist palliative care expertise when needed, and all staff providing this care should have undergone appropriate training. The guidelines are ambiguous about how palliative care should be integrated within stroke services, and no distinction between those patients who die in the acute stage and those who die in later stages of the disease pathway is made. In non-acute stroke, patients near the end of life have time to prepare for death, and professionals have an opportunity to assess needs, organise and implement appropriate interventions. In addition, the prevailing culture underpinning stroke care reflects a growing evidence-base for acute neurological intervention, patient activation and rehabilitation approaches, which may be difficult for staff to reconcile with palliative care. The transferability to stroke of palliative care concepts, which originate in cancer, may be problematic as recovery patterns, dying pathways, and the profile of patient problems and needs are likely to differ. An explanatory, theoretical account that describes the integration of palliative and stroke care from the perspectives of clinicians, patients and families is required to guide the development of practice and research.

## Methods

The aim of this study was to develop a programme theory to explain the integration of palliative and acute stroke care around the needs, experiences and preferences of patients and family members.

The integration of palliative care within a stroke context will involve a complex mix of multiple components such as patient assessment, psychological support, care planning and symptom control. Complex interventions should be represented by programme theories, comprising hypotheses which explain the impacts of components [16], and which, once tested, provide an evidence-base for clinical practice [17,18]. Approaches to defining programme theory which are limited to intervention inputs and outputs, for example logic models, can fail to capture the influence of contextual conditions which influence how the theory operates [19]. For example, different practical approaches to addressing stroke patients’ palliative care needs may need to be tailored to individual circumstances such as patient preferences and expectations, and should reflect the varying skills and capacities of individual clinicians for palliative care, and the settings within which they work. Experimental theory testing in a randomised controlled trial seeks to remove the influence of context. A realist approach to programme theory development and testing focuses on the contingent and cumulative nature of change, and reflects a more contingent view of ‘what works’ [20]. Located within critical realism, realist theories are described in terms of the contextual conditions and mechanisms of action that are activated or released through intervention, which cumulatively realise outcomes [21]. At their simplest level, interventions (such as components of palliative care) will, in the right conditions (context), change the thinking or behaviours (mechanisms) of clinicians, patients and others. It is these changes which, assuming that contextual factors remain supportive, cumulatively affect outcomes.

Different stakeholder groups will hold different views about theoretical explanations embedded within programme theory [16], and reports of realist theory development pay little attention to how different perspectives should be accommodated. This paper synthesises three sources of data collected from a programme of studies undertaken by the authors:

an investigation of palliative care needs in acute stroke (Study 1) [1],

an exploration of patient and family preferences and experiences of palliative care (Study 2) [2], and

group interviews with health professionals from three UK stroke services.

In doing so, we aim to produce an explanatory practice model to help clinicians meet the palliative and end of life care needs of patients and families through the integration of palliative care within acute stroke services.

In study 1, a consecutive cohort of acute adult stroke admissions (n=191) was assessed using the Sheffield Profile for Assessment and Referral to Care (SPARC) [22], which measures perceptions of needs across physical, social domains. Through the use of a structured assessment completed on average one week after stroke onset, study 1 provided a comprehensive overview of the range and intensity of problems. Study 2 comprised interviews that explored service experience, knowledge, preferences for care and perceptions of the future were conducted with 28 patients and 25 adult family members. The importance of excellent communication reinforced through inter-personal relationships between staff and families appeared to mitigate the difficulties associated with prognostic uncertainty. Although limited opportunities for engagement in clinical decision-making were identified, the data provide insights into the meanings that patients and family members attach to elements of the stroke service, and how these may impact on expectations and experience.

As the analytical purpose of the synthesis was building programme theory, sampling was purposive [23], focusing on the perspectives of those planning and delivering stroke services. To assure the theoretical transferability of our findings, our sampling strategy attempted to balance differences in stroke service design and perspectives across different professional groups. 29 staff from a range of professional groups (Table 1) across three hospital-based stroke services in the north of England participated in a group interview conducted in each clinical site. Although distinct clinical services, the three were connected through regional approaches to strategic service development in line with national stroke policy [24]. 

**Table 1:** Professional profile of group interview participants

	**Stroke service**			
**Professional group**	**Site A**	**Site B**	**Site C**	**All participants**
Clinical Psychology	1			1
Family advocacy	1			1
Health Care Assistant	1			1
Medicine	1			1
Occupational Therapy	1			2
Physiotherapy		1	1	2
Specialist stroke nursing	3		2	5
Speech and Language Therapy			1	1
Stroke unit nursing	2	3	2	7
Unknown (Did not wish to identify)	7	1	1	8
	n=17	n=5	n=7	N=29

Each group interview was facilitated by an experienced stroke researcher (CB) and an experienced qualitative researcher seconded to undertake this aspect of the study. Participants were provided with written study information by a lead stroke clinician within each service, and written informed consent was obtained at the start of each group interview. Group interviews drew on findings from both studies to explore the organisational and clinical barriers and facilitators to the development of palliative care provision in acute stroke. Each group was presented with a written summary of palliative care need, consisting of bar charts indicating the prevalence of reported needs as assessed by the SPARC (Study 1), with representative quotations relative to different need domains (Study 2). A semi-structured schedule was then used to guide participants to identify the clinical, professional and organisational issues pertinent to these needs. Interview topics included meanings of palliative care, including referral issues; recognition and assessment of palliative care needs and generalist capacity within the stroke service; the role of specialist palliative care within acute stroke; perspectives on working with families; and workforce and organisational development issues. Interviews, which ranged from 39 to 47 minutes, were audio recorded with the participants’ permission. Recordings of the group interviews were fully transcribed and managed in Atlas-Ti software.

To facilitate the synthesis across studies, each group interview was scrutinised by CB for potential mechanisms that characterised or explained the integration of palliative and acute stroke care. Mechanisms related to some type of change (or resistance to change) in staff knowledge, beliefs or behaviour at the interface between palliative and stroke care. Potential impacts of these mechanisms and contextual influences on changes were also recorded where present in the group interview transcript. Potential mechanisms were then summarised across the group interviews to reduce duplication across clinical or other topics. For example, data referring to whether palliative care was appropriate for a range of clinical problems highlighted a more general, abstract mechanism about the clinical legitimacy of palliative care. To complete the synthesis, mechanisms were then charted to juxtapose primary data and memos from Studies 1 & 2 alongside transcript excerpts from the group interviews with staff. An example for a mechanism referring to the clinical legitimacy of palliative care within stroke, and focusing on fatigue, is presented in Table 2. In keeping with realist approaches to theory building [25], the higher-level, abstract mechanisms are presented in this paper. 

**Table 2:** Example of charted synthesis across studies and group interviews with stroke service staff

**Clinical legitimacy – fatigue**			
**Group interviews**	**Study 1**	**Study 2**	**Synthesis**
*Staff exemplars* But ongoing symptoms like feeling tired or feeling weak, I’d probably say that feeling tired well that’s post stroke fatigue, that sort of thing that in the future will hopefully be alleviated with time. I mean there’s not many on there that I personally would say that would be, I’d never even thought of those. 2:17 (124:124)	*SPARC physical domain* Over 80% of sample reported some problems with ‘feeling weak’ or ‘feeling tired’. Nearly 40% rated these items as most severe.	*Patient exemplar* It was, I felt really, totally shattered initially… 5:2 (19:19)	The experience of fatigue is significant (in intensity and impact), although not clearly associated with palliative care.
	*SPARC psychological domain* Nearly 70% reported some problem with ‘feeling everything’s an effort’. Over 20% reported this item as most severe		Problems and needs relevant to palliative care are reported by patients and family members, although these tend not to be seen as palliative care when the patient is still being actively treated.
You’re alleviating any problems that they’ve got, like with pain, tiredness or discomfort but you’re not actively rehabilitating them. [2:24]	*Memo* Other than concerns about loss of independence and disability, these items were the most significant for participants across the SPARC assessment		
*Memo* Lack of awareness about intensity and range of problems within stroke population (as compared with cancer context)			

Ethical approval for this study was obtained from a NHS Local Research Ethics Committee, and appropriate governance approval to conduct the research obtained from participating NHS organisations. Approval included the use of transcript data in study reports and publications. Studies 1 and 2 were similarly approved, including the secondary analysis and publication of data.

### Findings

**Figure 1:**
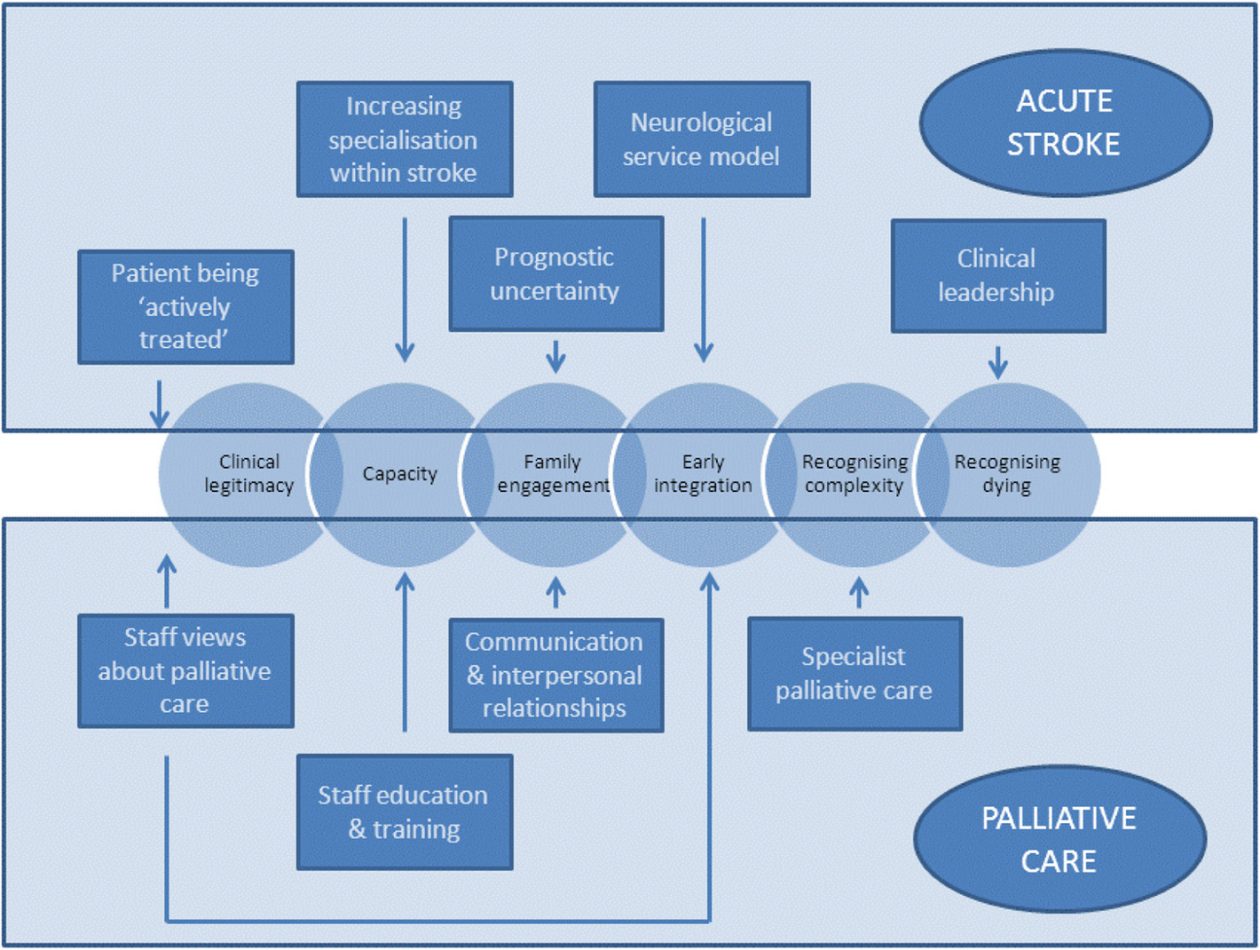
Theoretical map of the integration of Palliative and Acute Stroke Care.

Our data suggests that a programme theory that integrates palliative and acute stroke care should attend to six key mechanisms (Figure 1). Two cognitive mechanisms relate to the legitimacy of palliative care and individual capacity, whilst behavioural mechanisms relate to engaging with family, the timing of intervention, working with complexity and the recognition of dying. A range of clinical (whether patients are being ‘actively treated’ , and prognostic uncertainty) and service (leadership, specialty status and neurological focus) factors appear to influence how palliative care needs are attended to. Staff views, education and training, communication skills, supported by partnership working with specialist palliative care provide the basis for the integration of palliative and stroke care to occur.

#### Clinical legitimacy

Staff views on the range of problems reported by patients in Study 1 were consistent across the group discussions. Staff felt they could “*recognise these symptoms if you had interviewed X amount of cancer patients, not perhaps X amount of stroke patients* [2:20]. The four most significant problems reported by 80% of the study sample were ‘mobility problems’ , ‘feeling weak’ , ‘feeling tired’ , ‘being sleepy during the day time’ and ‘communication problems’. Although assessed as a psychological variable, 70% of the sample reported ‘feeling everything’s an effort’. Explanations of the reasons behind some problems such as tiredness and lethargy were generally discussed using language associated with stroke rather than palliative care practice. For example, tiredness was referred to as an effect of the care environment (such as noisy hospital wards) or post-stroke fatigue.

"I’m sure patients are tired but environmental factors are very big factors about not being able to get any rest on top of having a stroke [3,39]."

"…feeling tired well that’s post stroke fatigue [which] will hopefully be alleviated with time. [2:17]"

This attribution may be indicative of the perceived focus of palliative care within an acute stroke context: usually end of life care for patients with a poor prognosis.

"You’ve got the acutely ill patients, who’ve had massive haemorrhage or a massive infarct and you can tell that they’ve sustained significant damage and that they’re not going to recover from their stroke. [2:5]"

The physical consequences of stroke were associated with concerns about dependency and disability, with half of the participants reporting worries about the effects of stroke on families, and one in four felt they were likely to need more help than their family could provide. Approximately 50% experienced some form of psychological distress such as *‘anxiety’ , ‘low mood’ , ‘confusion’ , ‘poor concentration’* and ‘*loneliness’*. The SPARC data indicated that one in every four stroke patients had some concerns about death or dying. Whilst staff indicated the importance of helping patients and families to ‘cope’ psychologically with stroke consequences, whether this constituted palliative care was contested, with some participants preferring to use the term ‘supportive care’.

A tentative distinction between supportive and palliative care that emerged focused on the degree to which patients were being actively treated. Palliative care was viewed as supportive by some participants only when the overarching patient management strategy was active treatment such as rehabilitation. Palliative care was thought to relate mainly to the withdrawal of interventions.

"…palliative is almost like stepping back. You’re alleviating any problems that they’ve got, like with pain, tiredness or discomfort but you’re not actively rehabilitating them. [2:24]"

#### Capacity for palliative care

Features of the stroke service model include rapid access to a specialist service and the commencement of early interventions such as thrombolysis and rehabilitation. Rejection of the historical therapeutic nihilism around stroke has resulted in an increased patient acuity within an acute stroke service.

"In the past, we’ve only brought people to the acute Stroke Unit for assessment when they have been awake enough for assessment or else deemed ready for assessment. So if we saw them on the MAUs and they were unconscious or really quite poorly and expected to die, then we wouldn’t bring them down. But now, because … we are a specialist unit … we have a better turnover, faster discharges and fewer bed-blockers … those patients come down to the Stroke Unit where the staff are geared up for assessing patients and rehabbing the patients, to a very active environment. [3:30]"

Participants reported that the problems explored within Study 1 “are things that we ought to be able to manage within our [stroke] service” [3:40]. There was a recognition that the demand for specialist palliative care services was likely to increase, reinforcing the need to enhance the capacity for providing palliative care within the stroke service. Despite the increase in the numbers of patients with palliative care needs accessing stroke services, no evidence of a systematic approach to staff development was identified, with staff “learning on the job” [3:32].

Whilst a lack of staff development and training was identified, participants highlighted a number of opportunities that were felt to enhance the provision of palliative care. Case management, including the nomination of a key individual to liaise with family members, and to coordinate palliative care provision for individual patients was highlighted as having potential.

"… maybe a key person to be involved with the family and the patient. And if they’re happy with that key person, then they’ve got somebody familiar and they can feel they can trust them and give them the true, realistic, how the situation is, so that they can get the right input in. [1:12]"

The ability of the clinical environments to support the delivery of palliative care precipitated a considerable amount of staff discussion. The appropriateness of single rooms for those patients dying was equivocal, as “*isolating somebody in a cubicle in their last hours of life is very, very lonely”* [2:29]*.* Some participants felt uncomfortable about providing rehabilitation interventions, particularly when these required verbal encouragement, in close proximity to patients who were at the end of life.

"I do feel not particularly at ease if I know there’s somebody who is acutely unwell and I’m “come on Mrs Miggins, let’s stand up” you know. [3:34]"

In addition, the general business of the stroke service settings appeared to mitigate against a peaceful, restful and more appropriate environment for those with palliative care needs.

"I still think that there are environmental issues with an acute ward that, with the best will in the world, we have admissions coming in, happy wanderers, unhappy wanderers, muddled people, irritated people, in a relatively small space and a lot, you know we’ve got OTs, physios, speech and language, dieticians, pharmacists, medics, nurses, domestics, that’s a very busy environment and it isn’t conducive to rest. [3:44]"

#### Working with families

Honesty was valued by patients and families, even where prognosis was uncertain. However, staff were concerned about raising hope, and potentially false optimism.

"I think for relatives of these patients, nobody actually discusses the expectations and when you say we’re going to move them to the Stroke Unit, that can give false hope. [3:33]"

Although the importance of *“involving the patient if possible and the family from an early stage”* [3:20] was highlighted by staff, obstacles to meaningful engagement related both to the difficult nature of the information to be shared, particularly where prognosis is unpredictable, and to the processes of communication. Although preferences for both the depth and mode of information provision were variable amongst patients and families, they evaluated the quality of communication with staff primarily through inter-personal skills, rather than the prognostic accuracy of information that was imparted. However a need to balance professional uncertainty with patients’ and families’ needs for consistency in information was evident. Where patients were unlikely to recover from their stroke, then communication was perceived by staff to be both limited and difficult.

"… you’ve actually got nothing to say to them. It’s not that you’re writing them off it’s just that your mind is elsewhere"

"Yes, but you can see how people would perceive that as being “oh they’re withdrawing and just leaving us”"

"… I have tried to at least acknowledge “I’m sorry your father’s so ill and obviously we won’t be seeing him now” or something like that. It actually is quite difficult to say. [3:35]"

Whilst this may reflect the priorities within the stroke service, such as working to maximise patient recovery with rehabilitation as the predominant stroke service model, the potential negative impact of this difficulty on family members was recognised.

"…she said the thing that she found very difficult when visiting her father was … when it was clear that he wasn’t going to be alright, they all ignored the family … she felt that suddenly they weren’t spoken to whereas they could see other families having lots of meetings and talking and they were almost embarrassing to have on the ward. Probably because the therapists didn’t know what to say or do. [3:29]"

#### Early integration of palliative care

Staff recognised the importance of integrating palliative care before the final stages of dying, addressing “*the quality of life, not particularly end of life… even through the acute and the rehab phase, we would be looking at ‘not end of life’ palliative care*” [1:4]. Emphasising that palliative care need not be end-of-life care was mirrored in the use of palliative approaches to resolve symptoms such as fatigue:

"Because a lot of the patients who have the tiredness and fatigue are those who do really well in rehab, so you can’t say that they are in palliative care because they’re not. [3:54]"

Extending palliative care earlier than the terminal stages of a dying trajectory characterised palliation as a positive intervention strategy, shifting the emphasis from “*there’s nothing else we can do” whereas this is about “actually, there’s a lot we can do”* [3:8].

Where a patient may be labelled as ‘palliative’ by some members of a stroke team, active interventions such as physiotherapy was still appropriate. However this labelling was perceived as an obstacle to integrating palliative care alongside rehabilitation:

This difficulty perhaps reflects the active intervention strategies which characterise acute stroke care and where the provision of palliative care may be viewed as a failure of the rehabilitation process.

#### Recognising complexity

A distinction between generalist and specialist palliative care was drawn where staff felt there were “*very specific problems that we have with individuals having exhausted our repertoire”* [3:42], specifically in relation to symptom control and complex ethical issues. Examples included managing hydration and nutrition, and in exploring *“when do you stop? Have we made the right decision? … they* [palliative care specialists] *come along and they say “yes, yes, you should withdraw that, yes you’re not helping them, that should come down, you’re just prolonging the suffering” it helps because you think well, that’s not just my decision and they are experts at this”.* [2:12]

Stroke staff reported that access to specialist advice was useful in providing “*reassurance”* [2:13] and to “*support clinical decision-making*” [3:24]. Discussions about involvement of specialists in this area tended not to focus on partnership working through the addition of other, additional clinical perspectives or information. The focus was the provision of reassurance to the stroke team that appropriate decisions had been reached. This may reflect a lack of clarity about the clinical validity of specialist palliative care with regard to the needs of stroke patients:

"The difficulty with that is, there’s no specialism within the specialism. [1:9]"

#### Recognising dying

Reflecting advances in palliative care theory, difficulties in identifying a precise time point or phase when patients required palliative care were highlighted.

"At the moment I’ve got four patients on our floor who’ve been unconscious for three or four days and I’m sitting with the families saying “I just don’t know”. Now, would this be a time for palliative care? I don’t think so, because they may recover, but then again they may not. [3:36]"

As a consequence, decisions to formally assign a patient as requiring palliative care were “*very slow in the making. Almost to the point where the patient has almost passed away when the decision [to commence palliative care] is made”* [3:13]*.*

Data on decision-making focused primarily on who made decisions and the team context of decision-making, rather than on what basis decisions were made. Responsibility appeared to rest with the medical lead, although the decisions were couched in general terms, rather than an active decision to commence end of life care.

"…it’s the consultant, that actually says “we’re changing direction here”. Maybe from the information we’ve given him, but it’s very often them that take the lead in “OK, it’s time to go” and we can sway that decision, but I think ultimately it’s the consultant that will say “this is the direction we’re going in”. [3:22]"

Some participants felt that the organisation and delivery of stroke services often prevented doctors developing an in-depth understanding of individual patient’s circumstances and wishes.

"I wouldn’t say that they were best placed, but they are the ones that make the decisions, not necessarily the best person to make the decision. …they’re normally the people who see the patient least on ward rounds or whatever. And it’s actually the therapist, the nursing staff and the junior doctors that see the patient on a more regular basis and probably know the patient better than the actual consultants do. [3:25]"

It is important to note that currently this issue has not been reflected on by medical staff. Clearly other members of the multidisciplinary team view formal decision making about palliative care as a medical responsibility. However they appeared keen to highlight the different contributions that other professionals could make on the basis of their relationships with patients and family carers. Two factors appeared to facilitate decision-making: clinical experience and involving family members about palliative care decisions was highlighted.

"I think that’s individual to a therapist though, ones who haven’t got as much experience won’t want to make that decision, where the more senior people will say, because of the experience that they’ve got behind them and because of the experience they’ve got working in various teams. [2:9]"

## Discussion

This paper provides the first theoretical explanation of how palliative care and acute stroke care can be integrated around the needs and preferences of patients and families. The catastrophic impacts of stroke are well documented in the literature. The major emphasis of acute stroke care is on ensuring neurological recovery or stability, preventing complications and commencing early rehabilitation [13]. However patients and families also require access to palliative interventions that ameliorate negative disease sequelae, and support them at the end of life. The evidence base for the effectiveness of supportive strategies to address these issues in stroke is diffuse, and lacking in any theoretical integrity [26]. For example, relevant literatures will include, amongst other things, psychological care and emotional support, communication and information giving, carer and family support. With the emphasis of palliative care shifting from terminal, end of life care to supporting quality of life for patients with life-threatening illness such as stroke, palliative care may provide a new theoretical focus for enhancing practice in this area. This does not imply that palliative care is theoretically secure, as debate continues as to the natures of supportive, palliative and end of life care. However, quality of life in the face of life threatening illness may provide a mid-range theoretical focus around which different theory areas may be synthesised.

This paper highlights some contextual challenges associated with the integration of palliative and acute stroke care, although the clinical capacity to support patients and families through disease onset and progression is clearly evident. Improvements in the effectiveness of acute stroke care rightly focus the minds of clinical staff on neurological care and rehabilitation. For many, palliative care was primarily associated with the final stages of dying, and failure on the part of the clinical team. This may limit the potential for new insights to emerge from the synthesis of palliative care and other treatment modalities. A shift in thinking is required which acknowledges the potential benefits of earlier integration of palliative care for patients who have not reached the end of life.

Previous literature reviews examining the interface between palliative and stroke care have highlighted that few interventions are defined as ‘palliative’. National Clinical Guidelines for Stroke [5] reinforce the importance of access to expertise and the availability of skills, rather than the provision of specific interventions. An earlier review of the literature highlighted only one intervention study, a limited evaluation of the Liverpool Care Pathway [27], which suggested that the use of a protocol for end of life care improved some aspects of clinical care. Improvement in communicating poor prognosis to family members was more resistant to change. However, no information about interventions that may be applied earlier in the disease trajectory is available.

Data from one of the studies included in this paper provides detailed information about the range and intensity of patient-reported concerns within the acute stroke phase. The degree to which these concerns equate to problems that are the responsibility of statutory health services will be subject to debate. Our data indicate the significant concerns that patients and families may have for the future, including death and dying. Analysis of complaints sent to the Healthcare Commission for independent review between July 2004 and July 2006, showed that more than half (54%) of complaints about hospitals were about care surrounding a death. Specifically, “in many cases, families have received contradictory or confusing information from the different staff caring for their relative. Or, when they have compared the information they have received following a death, they have found discrepancies in what they have been told” [6 p17]. Policy and guidance highlight the importance of information provision, communication and decision-making within a multi-disciplinary context, and in partnership with patients and family to determine care preferences [7,28]. However implementation is inconsistent, particularly for patients whose recovery is uncertain [29]. Recognition of a stroke patient’s ‘dying’ status may be ambiguous [2], potentially resulting in over or under treatment and delaying initiation of general palliative care or referral to specialist palliative care. Older people generally may be disadvantaged in access to appropriate and acceptable services that meet their preferences and those of their family carers, who may also be older people [30]. There are problems in recognizing the process of dying and assigning an entry point to “end-of-life” is always going to be somewhat arbitrary [31]. Hypothesized models of typical dying trajectories linked to cancer, organ failure, frailty have not always been supported by empirical data [32,33], and the range of dying trajectories within acute stroke is unknown.

Inevitably, acute stroke onset presents a significant threat to patients and families, and these impacts are well documented in the literature. Whilst our data are confirmatory, they do provide some additional insights into how clinical care can be provided in a way that does not add further to distress. Notwithstanding the difficulties in accurately prognosticating outcome, although the majority of patients survive acute stroke, patients and their families have concerns about death and dying that do not appear to be related to prognosis. Opportunities to discuss and help make sense of these concerns are important to patients and families, and our data do not indicate that any lack of prognostic uncertainty should prevent these discussions from taking place. Honesty and excellent communication and inter-personal skills would appear to be central to the development of therapeutic relationships between patients, families and staff. Whilst it may not be possible for many concerns to be resolved by intervention, greater awareness and insights of patient and family concerns may mean that health care systems do not compound an already stressful situation. Practical steps identified by patients such as understanding how family networks operate around the patient, agreeing arrangements for communication, and helping patients and families make sense of their experience through, for example, keeping diaries, may all help in minimising the risks of additional negative experiences.

Our data demonstrate that the relationship between stroke and specialist palliative care tends to be reactive, confirming clinical decisions about palliation that have already been made by stroke clinicians. This may reflect the lack of evidence for specialist palliative care interventions for people affected by stroke, the increasing acuity of patients within acute stroke services, and the more general demand on specialist palliative care resources. Partnership working needs to shift from reactive support for clinical decisions, to more strategic collaboration that enhances the evidence base and care quality. New models of partnership working are required at both clinical and organisational levels, and importantly through collaborative research endeavour.

As a synthesis, the findings of this analysis may reflect limitations embedded in contributing data sources. These included survey and interview data obtained from one stroke service, and although some aspects of organisational context may have been specific, national audit data indicate that clinical practice was representative of that across the UK. Synthesis across studies also requires consideration of how contextual influences and matters of interpretation are addressed [34]. In this synthesis, data collection in the different studies was broadly underpinned by current frameworks for palliative care [9], which provided a consistent reference for interpretation. However we also attempted to synthesise alternative stakeholder views on palliative care within a stroke context. ‘Realist’ work requires a strong stakeholder focus to ensure that emerging theory addresses important issues, and produces useful findings [20]. However, little guidance is available to suggest how different perspectives should be managed within the process. As we aimed to produce a guiding framework for clinicians and service managers to sustain the integration of palliative care within stroke services, we ‘focused’ our synthesis through the perspectives of staff drawn from three stroke services. Whilst this should maximise the utility of our findings, we may have under-represented some issues which are important to other stakeholders, including patients and family members.

## Conclusion

This paper addresses an important gap in the literature by investigating the interface between stroke and palliative care from the perspectives of patients, family members and stroke service staff. Synthesis of three studies highlights a chain of mechanisms which cumulatively explain these may be integrated around the needs and preferences of patients and family members. Mechanisms relate to the legitimacy of palliative care and individual capacity, engaging with family, the timing of intervention, working with complexity and the recognition of dying. A range of clinical and service factors appear to influence whether these mechanisms operate, and consequently how palliative care needs are attended to. The beliefs of staff about palliative care, education and training, communication skills, supported by partnership working with specialist palliative care provide the basis for the integration of palliative and stroke care to occur. Our findings highlight difficulties in identifying the nature and purpose of palliative care in acute stroke services, including whether palliative care focuses on end of life care, or more general supportive interventions that could (or not) be combined with active treatment strategies. Practical difficulties in identifying when patients require palliative interventions should be the focus of further investigation.

## Competing interests

The authors declare that they have no competing interests.

## Authors’ contributions

CB and SP were involved in the study concept, design, analysis, interpretation of the data and drafting the manuscript. Both authors read and approved the final manuscript.

## Pre-publication history

The pre-publication history for this paper can be accessed here:

http://www.biomedcentral.com/1472-684X/11/22/prepub
